# Information Theoretic Methods for Future Communication Systems

**DOI:** 10.3390/e25030392

**Published:** 2023-02-21

**Authors:** Onur Günlü, Rafael F. Schaefer, Holger Boche, H. Vincent Poor

**Affiliations:** 1 Information Coding Division, Linköping University, 58183 Linköping, Sweden; 2 Chair of Information Theory and Machine Learning, Technische Universität Dresden, 01062 Dresden, Germany; 3 BMBF Research Hub 6G-Life, Technische Universität Dresden, 01062 Dresden, Germany; 4 Lehrstuhl für Theoretische Informationstechnik, TUM School of Computation, Information and Technology, Technical University of Munich, 80333 Munich, Germany; 5 CASA: Cyber Security in the Age of Large-Scale Adversaries Exzellenzcluster, Ruhr-Universität Bochum, 44780 Bochum, Germany; 6 BMBF Research Hub 6G-Life, Technical University of Munich, 80333 Munich, Germany; 7 Munich Center for Quantum Science and Technology (MCQST), Schellingstr. 4, 80799 Munich, Germany; 8 Department of Electrical and Computer Engineering, Princeton University, Princeton, NJ 08544, USA

It is anticipated that future communication systems will involve the use of new technologies, requiring high-speed computations using large amounts of data, in order to take advantage of data-driven methods for improving services and providing reliability and other benefits. In many cases, information theory can provide a fundamental understanding of the limits to the reliability, robustness, secrecy, privacy, resiliency, and latency of such systems. The aim of this Featured Special Issue has been to develop a collection of top information and coding theoretic results that provide insight into future communication and computation systems. 

The top-notch quality contributions to this Featured Special Issue consist of 11 articles, one of which is a review article. The topics touched upon include a multi-layer grant-free transmission method [1], a direct transform-coding approach that maps the delay-Doppler domain to the time domain [2], degree-of-freedom bounds for multi-antenna, multi-user, and frequency-selective interference channels with an instantaneous relay with or without coordination [3], new coded caching methods to reduce latency with user cooperation and simultaneous transmission [4], and a low-resolution downlink precoding method for multi-input single-output channels with orthogonal frequency-division multiplexing [5]. Furthermore, machine learning methods are discussed in the context of knowledge graphs for semantic communications [6] and in a review of the state-of-the-art coding methods for large-scale distributed machine learning [7]. Focusing on coding theory over rings, a new weight that extends the traditional Hamming weight used for algebraic structures is proposed and its properties are analyzed in [8]. Moreover, security aspects for future communication and computation systems are considered to analyze Gaussian wiretap channels with a jammer that overhears the transmissions [9], to propose new polynomial codes that enable straggler-tolerant secure matrix multiplication [10], and to illustrate the private-key rate regimes observed when reconstructing source sequences at another node with side information under privacy and security constraints [11]. It is expected that these contributions will have a significant impact on the applications of information and coding theory to future communication and computation systems.
